# Evaluation of Cardiac Troponin Levels in Individuals Aged 12–30 Years Following mRNA-1273 Vaccination: Results From a Randomized Placebo-Controlled Trial

**DOI:** 10.1093/ofid/ofag139

**Published:** 2026-03-21

**Authors:** Susan Pfeiffer, Margaret Rhee, Scott Striplin, Veronica Urdaneta, Walter Straus, Xiaowei Wang, Bethany Girard, Frances Priddy, Spyros Chalkias

**Affiliations:** Moderna, Inc., Cambridge, Massachusetts, USA; Velocity Clinical Research, Cleveland, Ohio, USA; Velocity Clinical Research, New Orleans, Louisiana, USA; Moderna, Inc., Cambridge, Massachusetts, USA; Moderna, Inc., Cambridge, Massachusetts, USA; Moderna, Inc., Cambridge, Massachusetts, USA; Moderna, Inc., Cambridge, Massachusetts, USA; Moderna, Inc., Cambridge, Massachusetts, USA; Moderna, Inc., Cambridge, Massachusetts, USA

**Keywords:** cardiac troponin I, COVID-19, COVID-19 vaccine, mRNA-1273, myocarditis

## Abstract

**Background:**

Myocarditis is a very rare event following COVID-19 vaccination, with highest risk observed in males aged 12–24 years. Cardiac troponin I (cTnI) is a cardiac-specific biomarker of myocardial injury but is not etiologically specific and may be elevated in the absence of cardiac pathology, including after vigorous physical activity. This study assessed cTnI levels following administration of mRNA-1273.712 vaccine versus placebo in participants aged 12–30 years.

**Methods:**

In this phase 4, observer-blind, randomized, placebo-controlled, crossover study, participants received mRNA-1273.712 50 µg and placebo 28 days apart. Cardiac troponin I was measured at baseline and 3 and 28 days after injection. The primary endpoint was the proportion of participants with elevated cTnI 3 days after injection. Safety was monitored throughout.

**Results:**

Of the 1000 participants randomized, 981 had evaluable cTnI results; most (>98%) maintained normal cTnI levels at all measured time points, including at 3 days postinjection. 18/981 participants had ≥1 elevated cTnI result at any study time point; 7 of those 18 had elevations at baseline prior to any injection. Of the remaining 11 participants with normal baselines and no prior elevations, 3 had elevated cTnI 28 days after receiving mRNA-1273.712 and 8 had elevations after placebo. 11/18 participants with elevated cTnI reported recent physical activity, and none exhibited clinical signs or symptoms of myocarditis or pericarditis. No new safety concerns were identified.

**Conclusions:**

Cardiac troponin I elevations were low overall and were observed after both placebo and mRNA-1273 administration. Vaccination with mRNA-1273.712 in adolescents and young adults was not associated with elevated cTnI.

## Teaser

This phase 4, randomized, observer-blind, placebo-controlled, crossover study assessed serum cardiac troponin I (cTnI) levels following administration of mRNA-1273 KP.2 variant-containing vaccine versus placebo in participants aged 12–30 years. Vaccination with mRNA-1273.712 in adolescents and young adults was not associated with elevated cTnI.

Myocarditis associated with SARS-CoV-2 infection has been reported since the early stages of the COVID-19 pandemic [1–3]. Myocarditis has also been observed after administration of both mRNA and non-mRNA COVID-19 vaccines [4–6]. Post-marketing surveillance of COVID-19 vaccines has demonstrated an increased risk of myocarditis and pericarditis following COVID-19 vaccination, with reported rates below 1 event per 10 000 doses administered [7, 8]. For the mRNA-1273 COVID-19 vaccine, the observed risk was highest after the second dose in the primary series, and in males aged 12–24 years, within 14 days after vaccination [8, 9]. Based on the US passive surveillance reporting, the rate of myocarditis after mRNA-1273 first-booster dose in young men (18–24 years) was 8.7 cases per 1 million doses administered [10]. This was considerably lower than the rate reported after the second dose of mRNA-1273 primary series in the same age group and time period (56.3 cases per million doses administered) [11]. Similarly, a cohort study from Nordic countries reported a higher incidence rate of myocarditis occurring within 28 days after the second primary dose (6.87 cases) versus the booster dose (1.95 cases) of mRNA-1273 in men aged 12–39 years per 100 000 individuals [12]. These observations indicate that myocarditis following mRNA-1273 vaccination has been reported most frequently after completion of the primary 2-dose series, with lower reported rates following subsequent doses administered under current vaccination schedules. These events were described as generally mild and often self-limiting, with resolution of symptoms within a few days under conservative management [7, 8]. However, the underlying pathogenesis and risk factors for vaccine-related myocarditis and pericarditis remain poorly understood [8, 9].

The estimated unadjusted incidence of myocarditis and/or pericarditis for the 2023–2024 vaccine formula (mRNA-1273.815 50 µg) is approximately 8 cases per million doses in individuals 6 months to 64 years of age and 27 cases per million doses in males 12–24 years of age within 7 days after vaccination [4]. The relative risk (RR) of myocarditis is more than 7-fold higher following SARS-CoV-2 infection (RR: 15, 95% CI: 11.09–19.81) than after mRNA COVID-19 vaccination (RR: 2.0; 95% CI: 1.44–2.65) [13]. The cardiac complications of symptomatic COVID-19 infection, including heart failure, arrhythmias, ischemic heart disease, and myocarditis, have been reported, particularly those with severe disease or underlying cardiovascular risk factors [14].

Cardiac troponin I (cTnI) is a serum biomarker used to assess myocardial injury and is routinely employed in the diagnosis of myocardial infarction due to its high sensitivity and specificity [15]. Elevations in cTnI provide evidence of cell degradation and allow identification of damaged cardiac tissue such as necrosis associated with myocarditis [16]. Elevated cTnI lacks high specificity for determining the underlying cause of the myocardial injury and can be elevated by various cardiac and noncardiac processes, including stress, exercise, rhabdomyolysis, and autoimmunity [16–18]. The relationship between elevated cTnI and nonpathogenic processes, such as vigorous physical activity, is well established [19, 20]. To date, cTnI levels have not been studied extensively in adolescents or young adults in the context of COVID-19 vaccination.

Here, we present the findings from a phase 4 placebo-controlled trial assessing cTnI levels following injection with mRNA-1273.712 or placebo in adolescent and young adult participants.

## METHODS

### Study Design and Participants

This was a randomized, observer-blind, placebo-controlled, crossover, phase 4 clinical study conducted across 24 centers in the United States to evaluate cTnI levels following administration of mRNA-1273.712 KP.2 variant–containing vaccine or placebo in adolescents and young adults (ClinicalTrials.gov: NCT06634797).

Eligible participants were male and female adolescents and young adults aged 12–30 years, inclusive. Participants who had a history of SARS-CoV-2 infection within 3 months prior to enrollment, had a history of myocarditis or pericarditis, had been acutely ill or febrile less than 72 hours prior to or at the screening visit or on day 1, or had conditions that may cause elevated cTnI were excluded. Full eligibility criteria are provided in the Supplementary Materials. Participants were randomized (1:1) to receive mRNA-1273.712 or placebo in a crossover design. Participants received either mRNA-1273.712 (50 µg) followed by placebo 28 days later (sequence 1) or placebo followed by mRNA-1273.712 50 µg 28 days later (sequence 2). Each participant received an injection on day 1 and day 29 according to their assigned sequence. The crossover study design allowed all participants to be vaccinated against COVID-19 consistent with recommendations, while allowing comparison of cTnI levels after vaccine versus placebo.

This study was conducted in accordance with the protocol and consensus ethical principles derived from international guidelines including the Declaration of Helsinki, Council for International Organizations of Medical Sciences international ethical guidelines, applicable International Council for Harmonisation Good Clinical Practice guidelines, and other applicable laws and regulations. The study protocol, informed consent forms, investigator brochure, and other relevant documents were reviewed and approved by an institutional review board prior to study initiation. All participants provided written informed consent prior to any study procedure.

### Objectives

The primary objective was to assess cTnI levels in participants who received mRNA-1273.712 or placebo. The primary endpoint was the proportion of participants with elevated cTnI levels at day 4 or day 32 (ie, 3 days after injection 1 or injection 2, respectively). Secondary objectives included further characterization of cTnI levels in both treatment groups by evaluating the proportion of participants with elevated cTnI levels at day 1 (baseline) and the proportion of participants with elevated cTnI at day 29 or day 57 (28 days following injection 1 or 2). Safety was also assessed as a secondary objective.

As an exploratory objective, cTnI levels were assessed on days 1, 4, 29, 32, and 57.

### Study Interventions and Procedures

mRNA-1273.712 is a lipid nanoparticle–encapsulated mRNA-based vaccine encoding the full-length spike protein of the SARS-CoV-2 KP.2 omicron subvariant stabilized in its prefusion conformation. This was the 2024/2025 formula that was licensed at the time the study was conducted. mRNA-1273.712 or placebo was administered by injection into the deltoid muscle.

Blood samples for cTnI assessments were collected on days 1, 4, 29, 32, and 57. Cardiac troponin I levels were measured using the Atellica® IM High-Sensitivity Troponin I (TnIH) assay (#10997840), a clinically validated, high-specificity assay intended for *in vitro* diagnostic use based on quantitative measurement of cTnI in human serum or plasma, using the Atellica® CI Analyzer. Sex-specific 99th percentile reference limits were used (>53.53 pg/mL for males and >38.64 pg/mL for females), with a lower limit of quantification of 3 pg/mL to ensure high sensitivity while maintaining reasonable specificity. Further details about the assay are provided in the Supplementary Materials. To aid interpretation of cTnI assessment, participants were instructed to record vigorous physical activities in an electronic diary (eDiary) during predefined study windows. The eDiary was completed for 4 consecutive days postinjection 1 (day 1), 4 consecutive days before and after injection 2 (day 29), and 4 consecutive days prior to the end-of-study (EOS) visit (day 57) and on the EOS visit. Participants were encouraged, but not required, to avoid vigorous physical activity in the 4 days before and after each injection. Further details on eDiary assessments are provided in the Supplementary Materials.

Safety data included monitoring and recording of unsolicited adverse events (AEs). Medically attended AEs (MAAEs), AEs of special interest (AESIs), serious AEs (SAEs), and AEs leading to study withdrawal and/or discontinuation of study intervention were recorded from day 1 through EOS. Because the reactogenicity of mRNA-1273 has been extensively characterized in previous studies [21, 22], solicited adverse reactions were not evaluated in the current study. Additional safety data included vital sign measurement before and after each injection, physical exam findings, pregnancies, concomitant medications, and nonstudy vaccines.

### Statistical Analyses

The sample size was considered sufficient to provide a descriptive analysis of the cTnI levels after study intervention (detailed in the Supplementary Materials). The randomization set included all randomized participants, analyzed by assigned injection sequence. The safety set comprised those who received at least 1 dose of study injection and was used for all safety analyses. The evaluable set included safety set participants without major protocol deviations or confounding factors and was used for cTnI analyses.

All cTnI analyses were descriptive and conducted longitudinally at the participant level. The number and proportion of participants with elevated cTnI levels, defined as values above the sex-specific 99th percentile threshold, at specified time points were summarized for each endpoint, together with 95% confidence intervals (CIs) using the Clopper–Pearson method. The Miettinen–Nurminen method was used to determine the 95% CI difference in proportions between mRNA-1273.712 and placebo groups. Changes in the proportion of participants with elevated cTnI from preinjection (day 1 or day 29) to postinjection time points within injection groups, along with corresponding 95% CIs, were calculated using the adjusted Wald intervals for paired data. Missing troponin values were not imputed and were excluded from analyses.

All safety analyses were descriptive and based on the safety set. All statistical analyses were performed using SAS 9.4 (SAS Institute, Cary, NC).

## RESULTS

### Participants

Participants were enrolled between October 2024 and January 2025. Overall, 997 of 1000 randomized participants received a study injection; the evaluable set included 491 of 500 participants (98.2%) in sequence 1 and 490 of 500 (98.0%) in sequence 2, respectively (Supplementary Figure 1).

Demographics and baseline characteristics were similar between sequence groups (Table 1). Overall, most participants were White (639/981 [65.1%]), were female (528/981 [53.8%]), and were not Hispanic or Latino (823/981 [83.9%]); the median (minimum, maximum) age was 18 (12, 30) years. Approximately 45% of participants were adolescents 12–17 years of age.

**Table 1.** Baseline Demographics and Characteristics (Evaluable Set)

	Sequence 1	Sequence 2
	mRNA-1273 + Placebo (n = 491)	Placebo + mRNA-1273 (n = 490)
Age, y		
Mean (SD)	19.6 (5.7)	20.0 (5.7)
Median (min, max)	18.0 (12, 30)	19.0 (12, 30)
Age group, y		
≥12 to ≤17, n (%)	233 (47.5)	212 (43.3)
≥18 to ≤30, n (%)	258 (52.5)	278 (56.7)
Sex, n (%)		
Female	261 (53.2)	267 (54.5)
Male	230 (46.8)	223 (45.5)
Race, n (%)		
White	313 (63.7)	326 (66.5)
Black/African American	130 (26.5)	106 (21.6)
Asian	19 (3.9)	14 (2.9)
American Indian or Alaska Native	1 (0.2)	3 (0.6)
Native Hawaiian or other Pacific Islander	1 (0.2)	1 (0.2)
Multiple	21 (4.3)	30 (6.1)
Other	3 (0.6)	7 (1.4)
Not reported	3 (0.6)	3 (0.6)
Ethnicity, n (%)		
Hispanic or Latino	66 (13.4)	79 (16.1)
Not Hispanic or Latino	416 (84.7)	407 (83.1)
Not reported	8 (1.6)	4 (0.8)
Unknown	1 (0.2)	0
BMI, kg/m^2^		
Mean (SD)	24.7 (5.7)	25.3 (5.6)
Median (min, max)	23.8 (12.6, 39.9)	24.5 (14.6, 39.9)

The evaluable set included all participants in the safety set that had no major protocol deviations or conditions/medications that impacted critical or key analysis data.

Abbreviations: BMI, body mass index; max, maximum; min, minimum.

### cTnI Levels

Of the 981 participants in the evaluable set, 963 (98.2%) maintained normal cTnI levels at all measured time points, including the prespecified primary endpoint assessment 3 days postinjection. Eighteen participants (1.8%) had at least 1 elevated cTnI measurement above the sex-specific 99th percentile reference limits. Some participants had elevated cTnI values at baseline prior to any study intervention, and some experienced a single postinjection elevation, while others had multiple elevations at different time points. In addition, several participants consistently had detectable cTnI concentrations above the assay's lower limit of quantification but within the normal reference range.

Cardiac troponin I results are summarized here in complementary analyses. The first describes all participants with at least 1 elevation at any study time point, categorized by baseline status. Subsequent analyses focus on participants with normal preinjection cTnI levels, those who reported physical activity, and those with elevations observed after vaccination.

#### cTnI Elevation by Baseline cTnI Status

Of the 18 participants with 1 or more elevated cTnI measurements, 7 had elevations at baseline prior to receiving any study injection (mRNA-1273.712 or placebo). Among the remaining 11 participants with normal baseline values and no prior elevations, 8 first exhibited cTnI elevation 28 days after receiving placebo, and 3 first exhibited elevated levels 28 days after receiving mRNA-1273.712. None of these 3 experienced elevations 3 days after mRNA-1273.712 administration, which was the prespecified primary endpoint.

#### Participants With Normal Preinjection cTnI

Among participants with normal cTnI values prior to injection (day 1 or day 29, or both), a small number exhibited elevated values 3 or 28 days after either injection 1 or injection 2; however, these elevations were not necessarily the first elevation experienced by the participant during the study.

One participant exhibited an elevation 3 days after receiving mRNA-1273.712 (Table 2). This elevation was not their first during the study, as elevated cTnI levels had also been observed at baseline and on day 4 (3 days after placebo administration). Because of the crossover design, elevations could occur following one or both injections within the same participant.

**Table 2.** Summary of cTnI Results in Participants With Normal Preinjection cTnI Levels

	mRNA-1273.712 (N = 938)	Placebo (N = 942)
Participants with normal cTnI at baseline (preinjection) and nonmissing cTnI 3 d after injection, N1	890	910
Participants with elevated cTnI 3 d after injection, n (%)^a^	1 (0.1)	0
Participants with normal cTnI at baseline (preinjection) and nonmissing cTnI 28 d after injection, N1	858	853
Participants with elevated cTnI 28 d after injection, n (%)^a^	4 (0.47)	8 (0.94)

Elevated cTnI was defined as >53.53 pg/mL in males and >38.64 pg/mL in females; the LLOQ for the cTnI assay was 3 pg/mL.

Abbreviations: cTnI, cardiac troponin I; LLOQ, lower limit of quantification.

^a^Percentage is based on N1.

Twelve participants with normal preinjection cTnI levels exhibited elevations 28 days after injection 1 or injection 2. The number of participants with 28-day postinjection elevations was numerically greater following placebo (n = 8) than after mRNA-1273.712 (n = 4). Overall, the occurrence of cTnI elevations was low and was observed after both placebo and mRNA-1273.712 administration.

#### cTnI Elevations and Physical Activity

Participants recorded vigorous physical activity in an eDiary at prespecified time points before and after each injection and before the EOS visit. Among the 18 participants who experienced at least 1 elevated cTnI value after any injection (mRNA-1273.712 or placebo), 11 reported engaging in vigorous physical activity within the 4-day window preceding the associated blood draw (Table 3). Several participants with multiple elevations also reported repeated episodes of physical activity during the study.

**Table 3.** Listing of Participants With Elevated cTnI Levels by Injection Sequence (Evaluable Set)

Age (years)/Sex	Day 1 (Preinjection 1)^b^	Day 4	Day 29 (Preinjection 2)	Day 32	Day 57 (End of study)	Elevated cTnI on Day 1 (Preinjection 1)	Elevated cTnI after mRNA-1273.712 with no prior elevation	Elevated cTnI after placebo with no prior elevation	Relevant physical activity reported in eDiary
**Sequence 1 (mRNA-1273+placebo)**									

12/F	< 3 pg/mL	< 3 pg/mL	< 3 pg/mL	< 3 pg/mL	**85 pg/mL**			Y	Y
16/M	4 pg/mL	< 3 pg/mL	4 pg/mL	< 3 pg/mL	**196 pg/mL**			Y	Y
17/F	**39 pg/mL**	27 pg/mL	19 pg/mL	20 pg/mL	22 pg/mL	Y			
16/F	7 pg/mL	6 pg/mL	**60 pg/mL**	22 pg/mL	7 pg/mL		Y		
14/M	**183 pg/mL**	**1588 pg/mL**	-	-	-	Y			Y
22/M	8 pg/mL	26 pg/mL	11 pg/mL	19 pg/mL	**380 pg/mL**			Y	
**Sequence 1 total, N^a^**	2	1	1	0	3	2	1	3	3
**Sequence 2 (placebo+mRNA-1273)**									

14/M	< 3 pg/mL	8 pg/mL	< 3 pg/mL	< 3 pg/mL	**144** pg/mL		Y		
13/M	51 pg/mL	26 pg/mL	**111 pg/mL**	**78 pg/mL**	30 pg/mL			Y	
19/F	**105 pg/mL**	27 pg/mL	**50 pg/mL**	**46 pg/mL**	10 pg/mL	Y			Y
29/M	4 pg/mL	4 pg/mL	**732 pg/mL**	**372 pg/mL**	**173 pg/mL**			Y	Y
12/F	**481 pg/mL**	13 pg/mL	< 3 pg/mL	< 3 pg/mL	< 3 pg/mL	Y			Y
17/F	**41 pg/mL**	**48 pg/mL**	34 pg/mL	33 pg/mL	**42 pg/mL**	Y			Y
15/M	25 pg/mL	39 pg/mL	46 pg/mL	27 pg/mL	**56 pg/mL**		Y		Y
28/F	32 pg/mL	25 pg/mL	**41 pg/mL**	**43 pg/mL**	23 pg/mL			Y	Y
13/M	< 3 pg/mL	< 3 pg/mL	**127 pg/mL**	< 3 pg/mL	< 3 pg/mL			Y	Y
26/M	< 3 pg/mL	< 3 pg/mL	**2630 pg/mL**	**3492 pg/mL**	**390 pg/mL**			Y	
16/M	**55 pg/mL**	38 pg/mL	46 pg/mL	41 pg/mL	35 pg/mL	Y			
16/M	**116 pg/mL**	**68 pg/mL**	45 pg/mL	**157 pg/mL**	45 pg/mL	Y			Y
**Sequence 2 Total, N^a^**	5	2	6	6	5	5	2	5	8

All values were calculated using the units pg/mL. Elevated cTnI was defined as >53.53 pg/mL in males and >38.64 pg/mL in females; the LLOQ for the cTnI assay was 3 pg/mL. Elevated cTnI levels for individual participants at each time point are indicated by bold font. Relevant physical activity was defined as activity that was reported during the eDiary collection window prior to the corresponding blood draw that yielded an elevated result.

¶Abbreviations: cTnI, cardiac troponin I; eDiary, electronic diary; LLOQ, lower limit of quantification.

^a^N summarizes the total number of participants (per injection sequence) who had an elevated cTnI or had reported recent physical activity.

^b^Baseline cTnI levels at day 1 (prior to any study injection).

#### cTnI Elevations 3 Days After Injection Regardless of Preinjection cTnI

The primary endpoint was the proportion of participants with elevated cTnI levels 3 days after injection 1 or injection 2 (day 4 or day 32). Among the 9 total participants with elevated cTnI 3 days after injection (Table 4), 8 had elevated cTnI at the corresponding preinjection time point (day 1 or day 29; mRNA-1273.712, n = 6; placebo, n = 2). The remaining participant, who received mRNA-1273.712, had normal preinjection cTnI level at day 29 but had previously exhibited elevated levels at both day 1 (preinjection) and again on day 4 after injection with placebo.

**Table 4.** Participants With Elevated cTnI Levels After Vaccination (Evaluable Set)

	mRNA-1273^a^ (N = 938)	Placebo^b^ (N = 942)
**3** **d postvaccination**		
Participants with nonmissing cTnI at prevaccination and 3 d postvaccination	898	916
Participants with elevated cTnI at prevaccination, n (%) [95% CI]^c^	8 (0.9) [0.4, 1.7]	6 (0.7) [0.2, 1.4]
Participants with elevated cTnI 3 d postvaccination, n (%) [95% CI]^c^	7 (0.8) [0.3, 1.6]	2 (0.2) [0.0, 0.8]
Difference, % (mRNA-1273–placebo) [95% CI]^d^	0.6 [−0.1, 1.4]	
Change in proportion of elevated cTnI from prevaccination to 3 d postvaccination, % [95% CI]^e^	−1.0 [−0.6, 0.4]	−0.4 [−1.0, 0.1]
28 d postvaccination		
Participants with non-missing cTnI at prevaccination and 28 d postvaccination	865	859
Participants with elevated cTnI at prevaccination, n (%) [95% CI]^c^	7 (0.8) [0.3, 1.7]	6 (0.7) [0.3, 1.5]
Participants with elevated cTnI 28 d postvaccination, n (%) [95% CI]^c^	6 (0.7) [0.3, 1.5]	9 (1.0) [0.5, 2.0]
Difference, % (mRNA-1273–placebo) [95% CI]^d^	−0.4 [−1.4, 0.6]	
Change in proportion of elevated cTnI from prevaccination to 28 d postvaccination, % [95% CI]^e^	−0.1 [−0.9, 0.6]	0.3 [−0.5, 1.2]

Elevated cTnI was defined as >53.53 pg/mL in males and >38.64 pg/mL in females; the LLOQ for the cTnI assay was 3 pg/mL.

Abbreviations: cTnI, cardiac troponin I; LLOQ, lower limit of quantification.

^a^The mRNA-1273 group was defined as participants who received mRNA-1273 as injection 1 on day 1 (sequence 1) and participants who received mRNA-1273 as injection 2 on day 29 (sequence 2).

^b^The placebo group was defined as participants who received placebo as injection 1 on day 1 (sequence 2) and participants who received placebo as injection 2 on day 29 (sequence 1).

^c^95% CI was calculated using the Clopper–Pearson method.

^d^95% CI was calculated using the Miettinen–Nurminen method.

^e^Changes in the proportion of elevated cTnI were calculated as the proportion of elevated cTnI at specified time point—proportion of elevated cTnI at day 1 or day 29 (preinjection 1 or preinjection 2) within each injection group. 95% CIs were calculated using the adjusted Wald interval for paired data, where the preinjection and postinjection cTnI levels from the same participant are paired.

No notable differences were observed in the proportion of participants with elevated cTnI at 3 days postinjection between the 2 injection groups (difference: 0.6% [95% CI: −0.1%, 1.4%]). Similarly, there were no increases from preinjection baseline in proportion of participants with elevated cTnI levels after either mRNA-1273.712 injection (−0.1% [95% CI: −0.6%, 0.4%]) or placebo injection (−0.4% [95% CI: −1.0%, 0.1%]).

### Safety

In sequence 1, unsolicited AEs through EOS were reported in 1.2% of participants following mRNA-1273.712 injection and 1.1% of participants after placebo (Table 5); none were considered related to study injection by the investigator. All unsolicited AEs reported were MAAEs. Two AESIs were reported: 1 participant in the mRNA-1273.712 group with a medical history of generalized tonic-clonic seizures (not disclosed during screening) experienced a seizure leading to discontinuation of study injection and 1 participant in the placebo group experienced atrial fibrillation. Both events were assessed as unrelated by the investigator. One participant with elevated cTnI had a mildly abnormal electrocardiogram following mRNA-1273.712 administration; this event was also considered unrelated by the investigator. No fatal events, SAEs, severe unsolicited AEs, or AEs leading to discontinuation were reported in either group following injection.

**Table 5.** Summary of Unsolicited Adverse Events After Vaccination (Safety Set)

	Sequence 1		Sequence 2	
	mRNA-1273 + Placebo (n = 500)		Placebo + mRNA-1273 (n = 497)	
Adverse Events	mRNA-1273 (n = 500)	Placebo (n = 460)	Placebo (n = 497)	mRNA-1273 (n = 454)
Regardless of relationship to vaccination, n (%)				
All	6 (1.2)	5 (1.1)	9 (1.8)	10 (2.2)
SAE	0	0	0	1 (0.2)
MAAE	6 (1.2)	5 (1.1)	9 (1.8)	10 (2.2)
Leading to study discontinuation	0	0	1 (0.2)	0
AESI	1 (0.2)	1 (0.2)	0	0
Related to study vaccination, n (%)				
All	0	0	0	0
SAE	0	0	0	0
MAAE	0	0	0	0
Leading to study discontinuation	0	0	0	0
AESI	0	0	0	0

The safety set included all randomized participants who received at least 1 dose of study vaccination.

Abbreviations: AESI, adverse event of special interest; MAAE, medically attended adverse event; SAE, serious adverse event.

In sequence 2, unsolicited AEs through EOS were reported in 2.2% and 1.8% of participants following injection with mRNA-1273.712 and placebo, respectively (Table 5). All were MAAEs, and none were considered related to study injection by the investigator. One participant experienced nonserious AEs of rash and urinary tract infection after placebo injection, both leading to discontinuation of study injection; neither event was assessed as related to study injection by the investigator. One SAE of suicidal ideation occurred in a participant with a history of major depressive disorder after receiving mRNA-1273.712; this event was considered unrelated to study vaccination. No fatal events, AESIs, or severe unsolicited AEs were reported in either group.

## DISCUSSION

In this phase 4, placebo-controlled study in healthy adolescents and young adults aged 12–30 years, most participants maintained normal cTnI levels throughout follow-up, and few participants exhibited elevations, including at day 4 or day 32 (3 days after injection). Some elevated cTnI values were present at baseline before any study injection and others occurred at later study time points (>28 days postinjection). Among participants with normal baseline (Day 1) cTnI, no elevations occurred within 3 days of mRNA-1273.712 administration. Among those with normal preinjection (day 1 or day 29, or both) cTnI levels, 1 participant had an elevation 3 days after injection (mRNA-1273.712) and 12 had elevated values 28 days after injection (8 after placebo and 4 after mRNA-1273.712). Overall, cTnI elevations were uncommon, occurred after both mRNA-1273.712 and placebo, and were sometimes present prior to any study intervention.

A post-authorization review of mRNA-1273 based on 2 years of use estimated that the risk of myocarditis was highest within <7 days following vaccination (observed-to-expected [OE] ratio, 1.67; 95% CI, 1.56–1.78), especially among young individuals aged 12–40 years (OE, 3.89; 95% CI, 3.50–4.32) [23]. In the present study, no deaths or SAEs related to mRNA-1273.712 were reported, and no new safety concerns were identified. The infrequent and transient cTnI elevations observed in this study were not clinically meaningful.

None of the participants in the study, including 18 participants with elevated cTnI levels, reported signs, symptoms, or AEs consistent with standardized case definitions for myocarditis or pericarditis [24]. Recent physical activity was noted in 11 of 18 participants with elevated cTnI levels, and the elevations in these participants were not temporally associated with mRNA-1273.12 vaccination, suggesting that physical activity may have contributed to the observed elevations. Physical activity is a well-recognized nonpathologic driver of cTnI release [19, 20], with the type, duration, and intensity of exertion affecting detectable cTnI levels in otherwise healthy individuals [25, 26].

To our knowledge, this is one of the first studies to assess post-mRNA vaccination cTnI levels in healthy adolescents and young adults, a population at highest risk for COVID-19 vaccine-associated myocarditis [27, 28]. By incorporating cTnI measurements, this analysis expands prior safety evaluations of mRNA-1273 [21, 22]. Although cTnI lacks specificity for determining the cause of myocardial injury, cTnI elevations provide evidence of cell degradation and allow identification of damaged cardiac tissue [16, 17]. The current findings do not provide evidence of subclinical cardiac injury following mRNA vaccination and add to a growing body of evidence regarding the cardiac safety of mRNA-based COVID-19 vaccines in populations at increased risk for myocarditis.

A key strength of this study is its crossover design, which allowed comparisons of cTnI elevation before and after mRNA-1273 vaccination versus placebo. Limitations include the predominantly White, US-based participant population, which may limit generalizability. To better characterize the fluctuation of cTnI levels throughout the study, additional analyses were added to supplement the primary endpoints. Longitudinal summaries of all cTnI values for individual participants with at least 1 cTnI elevation during the study were included and assessed in the context of temporally relevant physical activity data collected in the eDiary. Interpretation of elevated values in healthy adolescents and young adults remains challenging due to limited data on age-specific reference ranges. Despite these limitations, there is no indication of any relationship between troponin I elevations and the administration of mRNA-1273.712.

In conclusion, this phase 4 study found that a single 50 μg dose of mRNA-1273.712 was not associated with elevated cTnI levels. No cases of myocarditis or pericarditis were reported or identified, including among participants with elevated cTnI levels, and vigorous physical activity was reported in 11 of the 18 participants with cTnI elevations. These findings support the overall favorable benefit-risk profile of mRNA-1273 vaccination in younger populations, including those at highest risk for vaccine-associated myocarditis.

## Supplementary Material

ofag139_Supplementary_Data

## Supplementary Data

Supplementary materials are available at Open Forum Infectious Diseases online. Consisting of data provided by the authors to benefit the reader, the posted materials are not copyedited and are the sole responsibility of the authors, so questions or comments should be addressed to the corresponding author.
