# Supplementary material for: Evaluation of Cardiac Troponin Levels in Individuals Aged 12–30 Years Following mRNA-1273 Vaccination: Results From a Randomized Placebo-Controlled Trial
Source: Open Forum Infect Dis. 2026 Mar 21;13(4):ofag139. doi: 10.1093/ofid/ofag139 (PMC13070467; doi:10.1093/ofid/ofag139)
Supplement: ofag139_Supplementary_Data [file ofag139_supplementary_data.docx]

# Supplementary Materials

## Eligibility Criteria

### Inclusion Criteria

Participants were eligible to be included in the study only if all of the following criteria applied:

1. At least 12 through 30 years of age (inclusive) at the time of signing the informed consent (screening visit)
2. Investigator’s assessment that participant understood and was willing and physically able to comply with protocol-mandated follow-up, including all procedures
3. Capable of giving signed informed consent which included compliance with the requirements and restrictions listed in the informed consent form and in the study protocol
4. Assigned female and/or male at birth

Contraceptive use by participants should have been consistent with local regulations regarding the methods of contraception for those participating in clinical studies

- Participants who could become pregnant
  - A participant who could become pregnant was eligible to participate if they were not pregnant or breast/chestfeeding and one of the following conditions applied
    - Was a person of nonchildbearing potential as defined in the Contraceptive and Barrier Guidance
    - Was a person of childbearing potential and fulfilled all of the following criteria
      - Had a negative highly sensitive pregnancy test on the day of injection prior to injection (day 1)
      - Had been using a highly effective or effective contraceptive method as described in Contraceptive and Barrier Guidance or had abstained from all activities that could lead to pregnancy for at least 28 days prior to the first injection (day 1). The Investigator evaluated the potential for contraceptive method failure (eg, noncompliance, recently initiated) in relationship to the first injection
      - Had agreed to continue adequate contraception through end of study
  - The Investigator was responsible for review of medical history, menstrual history, and recent sexual activity to decrease the risk for inclusion of a participant with an early undetected pregnancy

### Exclusion Criteria

Participants were excluded from the study if any of the following criteria applied

1. History of anaphylaxis or severe hypersensitivity reaction requiring medical intervention after receipt of any mRNA vaccine or therapeutic or any components of an mRNA vaccine or therapeutic
2. Had a known history of SARS-CoV-2 infection within 3 months prior to enrollment.
3. Had a documented history of myocarditis or pericarditis
4. Was acutely ill or febrile (temperature ≥38.0 °C/[100.4 °F]) less than 72 hours prior to or at the screening visit or day 1. Participants meeting this criterion could be rescheduled within the visit window and would have retained their initially assigned participant number
5. Had known conditions that may cause elevated cardiac troponin I (cTnI)

- Cardiac disease/conditions including rhythm disorders and congenital heart disease
- Diabetes
- Uncontrolled hypertension (defined as systolic blood pressure >140 mm Hg or diastolic blood pressure >90 mm Hg)
- Alcohol or substance abuse
- Kidney disease
- Severe obesity, defined as body mass index (BMI) ≥40 kg/m^2^ (>20 years) or severe obesity defined as BMI for sex and age ≥120% of the 95th percentile (BMI ≥35 kg/m^2^)
- Other conditions including infiltrative diseases (amyloidosis, sarcoidosis, hemochromatosis); respiratory conditions (chronic lung disease, pulmonary embolism with right ventricular dysfunction); inherited diseases (Duchenne muscular dystrophy); myocardial injury or trauma within 1 month of screening (cardiac surgery, chest wall trauma, drug toxicity [eg, adriamycin, 5-fluorouracil); autoimmune diseases that can cause inflammation or direct damage to the heart (systemic lupus erythematosus, rheumatoid arthritis, scleroderma, sarcoidosis, polyarteritis nodosa); miscellaneous (rhabdomyolysis within 1 month of screening)

1. Had symptomatic acute or unstable chronic disease requiring medical or surgical care, to include significant change in therapy or hospitalization for worsening disease, at the discretion of the Investigator
   - Clinically unstable was defined as a diagnosis or condition requiring changes in management or medication within the 60 days prior to screening and includes ongoing workup of an undiagnosed illness that could lead to a new diagnosis or condition
2. Had a medical, psychiatric, or occupational condition that could pose additional risk as a result of participation, or that could interfere with safety assessments or interpretation of results according to the Investigator’s judgment
3. Reported history of congenital or acquired immunodeficiency (eg, HIV), immunosuppressive condition or immune-mediated disease, asplenia, or recurrent severe infections disease
4. Had a history of Guillain-Barré syndrome
5. Had coagulopathy or bleeding disorder considered a contraindication to intramuscular injection or phlebotomy
6. Had a history of malignancy within the past 5 years (excluding nonmelanoma skin cancer)
7. Reported receipt of the following
   - COVID-19 vaccine within 3 months prior to the first injection or planned to receive a COVID-19 vaccine at any time during the study (except for study intervention)
   - Any other licensed vaccine within 28 days before the study injection or planned receipt prior to end of study
   - Systemic immunosuppressants or immune-modifying drugs for >14 days in total within 6 months prior to screening (for corticosteroids ≥10 mg/day of prednisone equivalent) or is anticipating the need for immunosuppressive treatment at any time during participation in the study
   - Systemic immunoglobulins or blood products within 3 months prior to the screening/baseline visit or planned receipt during the study
8. Had donated ≥450 mL of blood products within 28 days prior to the screening visit or planned to donate blood products during the study
9. Had participated in an interventional clinical study within 28 days prior to the screening visit or planned to participate in an interventional clinical study of an investigational vaccine or drug while participating in this study
10. Was an immediate family member or household member of study personnel, study site staff, or Sponsor personnel

### Prior COVID-19 Vaccination History

Information on prior COVID-19 vaccination was collected at screening as part of participant medical history and concomitant medication reporting. Prior COVID-19 vaccination occurring more than 3 months before enrollment was not an inclusion or exclusion criterion for study participation and was not a prespecified variable for analysis.

Consistent with real-world use conditions at the time the study was conducted, most participants were expected to have prior exposure to SARS-CoV-2 spike protein through vaccination and/or natural infection. The study was not designed or powered to evaluate the incidence of myocarditis or pericarditis by prior vaccination status, nor to compare cardiac troponin I levels across subgroups defined by prior COVID-19 vaccination history.

## eDiary Assessments

Vigorous physical activities were defined as activities that required hard physical effort and made the participant breathe much harder than normal, because these types of activities can elevate cTnI levels^16^. The eDiary did not include all possible vigorous physical activities; instead, it contained the following selection of activities typical for the target age group (12-30 years) that could elevate cTnI:

- Jogging/running
- Swimming/water exercise
- Playing an organized sport
- Riding a bicycle
- Lifting weights/using a stationary weight machine
- Heavy manual labor

Depending on when study injections or the end-of-study (EOS) visit were scheduled to be completed, participants were instructed to complete the eDiary for 4 consecutive days following Injection 1 (Day 1), for 4 consecutive days before and after Injection 2 (Day 29), and for 4 consecutive days prior to the EOS visit (Day 57), as well as on the day of the EOS visit. Participants were encouraged, but not required, to avoid vigorous physical activities, when possible, for 4 days before and after each injection.

## High-Sensitivity Troponin I Assay

The Atellica^®^ IM High‑Sensitivity Troponin I (TnIH) assay (#10997840) was used according to the manufacturer’s instructions. The assay is intended for in vitro diagnostic use based on the quantitative measurement of cardiac troponin I (cTnI) in human serum or plasma (lithium heparin) using the Atellica^®^ CI Analyzer. The Atellica IM TnIH assay is a 3-site sandwich immunoassay using direct chemiluminometric technology. The Lite Reagent comprises a conjugate whose architecture consists of a proprietary acridinium ester and a recombinant anti-human cTnI sheep Fab covalently attached to bovine serum albumin for chemiluminescent detection. The solid phase reagent was streptavidin-coated magnetic latex particles (0.45 mg/mL) with 2 bound biotinylated (mouse and sheep) monoclonal anti-troponin I antibodies in buffer, each recognizing a unique cTnI epitope. A direct relationship exists between the amount of troponin I present in the patient sample and the amount of relative light units detected by the system. A sample volume of 100 µL was used for a single determination. A sample at a volume of 100 µL was dispensed into a cuvette. Solid phase (130 µL) and Lite Reagent (80 µL) were then dispensed and incubated for 8 minutes at 37 °C. Cuvette was separated, aspirated, and washed using Atellica IM Wash. Atellica IM Acid and Atellica IM Base (300 µL each) were dispensed to initiate the chemiluminescent reaction. Results were reported in pg/mL, using the measuring interval for serum and plasma of 2.50-25,000.00 pg/mL.

## Sample Size Determination

The sample size was considered sufficient to provide a descriptive analysis of the cTnI levels after study intervention. The sample size was determined to provide 90% power to observe at least 1 participant who received mRNA-1273.712 (injection 1 or 2) with an elevated cTnI level at a true rate of 0.3%, allowing for approximately 10% exclusion of participants from the mRNA-1273.712 group (injection 1 or 2) in the evaluable set due to reasons such as early dropout, and major protocol deviations that impact key analysis data.

## Supplementary Figure 1. Participant disposition.


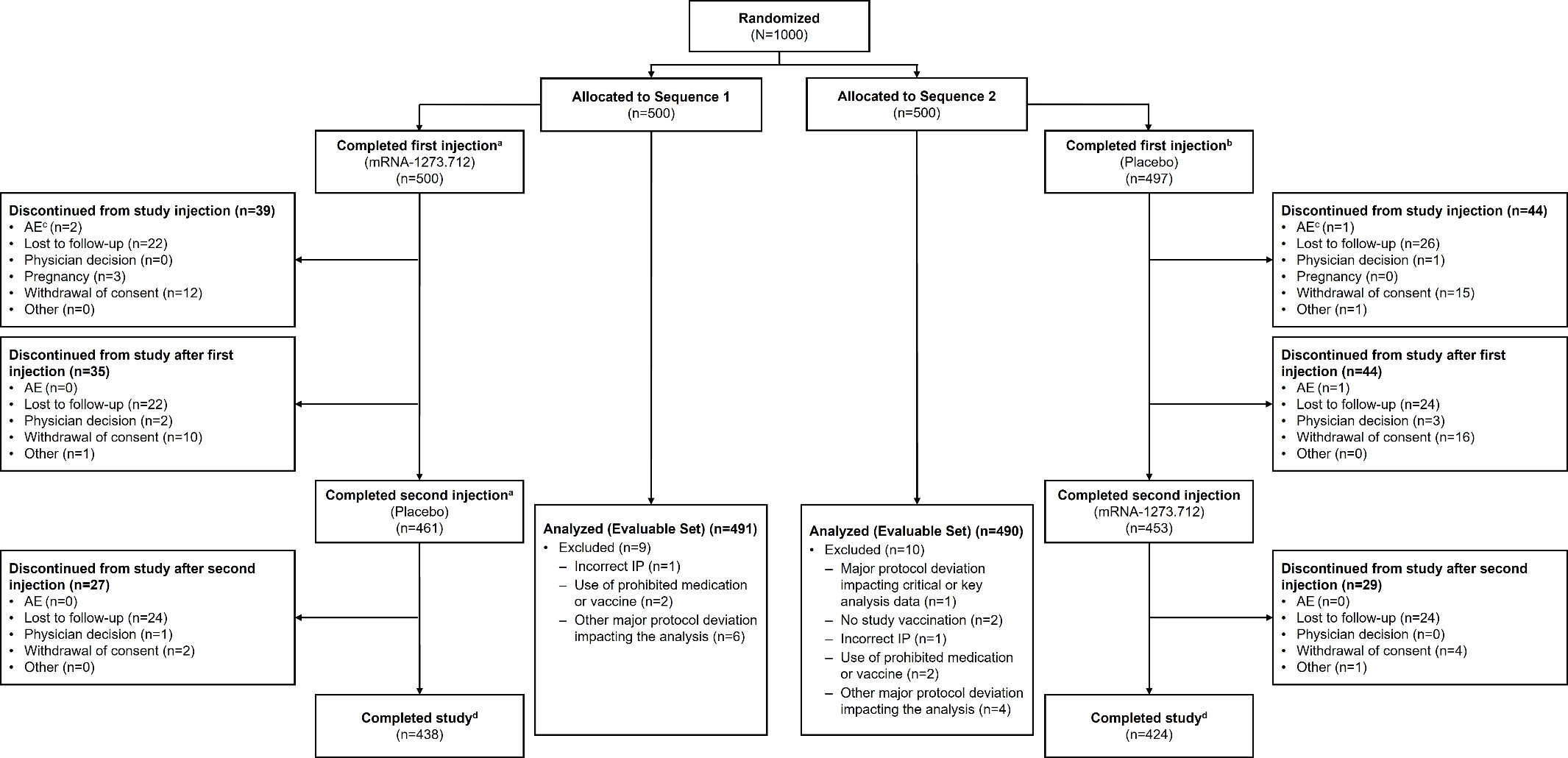


AE, adverse event; IP, investigational product

^a^Includes 1 participant who erroneously received placebo followed by mRNA-1273.712 for the first and second injections, respectively, due to a dosing error.

^b^Includes 1 participant who erroneously received mRNA-1273.712 for the first injection due to a dosing error.

^c^One participant initially had their second dose delayed due to an AE of abnormal electrocardiograph. Following repeat testing that was normal, the participant was scheduled for the second injection but failed to return. The reason for discontinuation of study injection was reported as AE, and the reason for discontinuation from the study was reported as physician decision, due to the participant’s noncompliance with the visit schedule and eDiary completion.

^d^Participants were considered to have completed the study if they completed the last scheduled procedure on day 57. Two participants were screen failures who were randomized in error. These 2 participants were not counted as either completed or discontinued from the study.
